# Exploring paramedics’ lived experiences in confrontation with patients’ death during missions: a phenomenological study

**DOI:** 10.1186/s12873-024-01042-6

**Published:** 2024-07-12

**Authors:** Milad Ahmadi Marzaleh, Mahmoudreza Peyravi, Esmaeil Ahmadi, Iman Shakibkhah, Hossein Armin, Hadi Mahmoodi, Hossein Avazaghaei

**Affiliations:** Department of Health in Disasters and Emergencies, School of Health Management and Information Sciences, Shiraz University of Medical Sciences, Shiraz, Iran

**Keywords:** Paramedic, Lived experiences, Phenomenology, EMT, EMS, Prehospital personnel

## Abstract

**Background:**

Emergency personnel are the first line of emergency response systems to respond to emergencies; in essence, they are usually exposed to a wide range of physical and psychological problems. Accordingly, the current study aimed to clarify the lived experiences of paramedics when exposed to Patients’ Deaths during their missions in 2023.

**Methods:**

This study was carried out using a qualitative approach and interpretative phenomenology from January 9, 2022, to September 21, 2023. The research was performed in Fars, Alborz, and Isfahan provinces in Iran. Data were gathered using semi-structured interviews with 17 male emergency personnel (both from the emergency medical service and Red Crescent). The obtained data were analyzed utilizing Smith’s approach to clarify the lived experiences of emergency responders when facing deaths in various incidents in Iran.

**Results:**

Seventeen emergency personnel with the age range of 24–60 (average = 39) years and with a history of confronting patients’ deaths during their services were interviewed. Their lived experiences of being exposed to patients’ deaths during the emergency response in Iran were classified into three main themes: psychological and emotional status, personality, disposition, and behavior status, and mental and physical status. Sub-themes such as psychological and emotional problems, mental and physical problems, and sub-subthemes such as anxiety, stress, decreased appetite, irritability, insomnia, forgetfulness, and fatigue were also noted within the main themes.

**Conclusion:**

While emergency personnel work diligently to save the lives of patients, the current study demonstrated that they were susceptible to multiple psychological, emotional, and physical problems, which potentially affect their lives outside of the workplace and make them more vulnerable to related physiological and psychological diseases. It is recommended that policymakers and clinical educators make ways to prevent these problems and provide emergency personnel with physical, psychological, and emotional support.

**Supplementary Information:**

The online version contains supplementary material available at 10.1186/s12873-024-01042-6.

## Introduction

Given the increasing trend in the rate of disasters worldwide, the need for emergency response personnel in emergency situations has increased [1]. For example, in England, for the period 2014–2015, there were over 9 million emergency calls requesting an ambulance, which is an increase of more than 500,000 compared to the previous year [2]. Emergency Medical Technicians (EMTs) play a critical role in providing medical care to critically ill patients [3]. All healthcare providers face challenges when providing care to patients, and in the pre-hospital field, emergency medical services (EMS) providers are no exception to this rule [4]. While emergency personnel make every effort to save the lives of patients, they are exposed to numerous physical, psychological, and emotional injuries, such as witnessing distressing scenes, the cries of dying patients, and seeing individuals with traumatic injuries, all of which can have a profound impact on the mental, psychological, and physical well-being of pre-hospital and paramedic personnel [5].

Research indicates that pre-hospital care providers, in comparison to the general population, have a higher risk of physical problems such as hypertension, obesity, and high cholesterol, as well as mental and psychological problems [5, 6]. This is particularly relevant as larger-scale disasters can lead to more injuries among emergency medical technicians (EMTs). The prevalence of post-traumatic stress disorder (PTSD) resulting from the experience of traumatic events in the general population is 10–15%, but this rate is higher among emergency response personnel, with a prevalence of 18.2% among German firefighters [7]. Various studies conducted on emergency medical service (EMS) personnel who have been involved in various incidents, such as airplane crashes, in Iran have shown that the prevalence of anxiety and stress in these individuals ranges from 17 to 25%, and it is estimated to be 16.73% in EMS personnel responding to earthquakes [5].

The identification of victims by the responding individual can exacerbate psychological injuries, and risk factors such as the age of the victim, exposure to horrifying injuries or death, and exposure to unpredictable dangers can increase the psychological and emotional injuries of pre-hospital and emergency personnel [7]. Most emergency personnel believe that they experience more stress when providing emergency services to the injured children [8]. Additionally, research has shown that pandemic conditions and the prevalence of viral diseases such as COVID-19 have put emergency responders under various psychological pressures, such as the use of protective clothing and N95 masks, exposure to the virus, and possibility of transmitting the virus to their families. A study conducted on the family members of 5 hospital staff in China showed that 37.7% of them experienced stress, and 29.35% of them had symptoms of depression [9]. A less understood topic is the lived experience of the personnel who, as emergency responders, were responding to disasters and pandemic conditions and were exposed to numerous traumatic deaths [10].

Based on the literature reviewed above, although the importance of the complications that emergency service providers might face during their mission have been well addressed the phenomenological aspects of these problems seem yet to be known. So, the aim of this study was to investigate the phenomenological experiences of emergency personnel from two organizations in Iran, the Pre-hospital Emergency Medical Service, and the Red Crescent, who have confronted multiple deaths in their missions and have witnessed many patients’ deaths. This study paves the ground for insights into a less-explored area of research: the lived experiences of emergency personnel who have a history of being exposed to patient deaths during their missions.

## Methods

This is a qualitative phenomenological interpretive study conducted from January 9, 2022, to September 21, 2023. The research was carried out in Fars, Isfahan, and Alborz provinces in Iran. Data were collected through interviews with the personnel from emergency response units, including emergency medical services (EMS) and the Red Crescent. The data were analyzed using Smith’s approach to elucidate the lived experiences of these personnel when encountering deaths in various incidents in Iran.

### Participants

The criteria for participant inclusion in the interviews were as follows:

1. Having experienced encounters with death.
2. Holding academic qualifications.
3. Possessing the physical and mental capacity to participate in interviews.
4. Having a work history of more than five years in emergency response systems.

The criteria for participant exclusion from the study were as follows:

1. Lack of willingness to participate.
2. Failure to meet any of the specified participation criteria.

Based on the given criteria 17 male participants have been included in this study to answer one semi-structured interview. A purposeful sampling method was employed, where individuals meeting the specified criteria were identified. After explaining the research objectives and importance to these individuals and obtaining their consent, we performed the interviews. Each interview was assigned a unique code, such as #1, #2, and so on, to ensure data confidentiality. The names of the individuals were not recorded in the demographic forms.

### Data collection

During interviews with the emergency personnel, both in-depth and semi-structured questions were asked regarding their experiences when encountering deaths. These encounters occurred during hospital transfers when responding to high-fatality incidents like natural disasters, and other relevant scenarios. Sample questions included: “What has been your experience when encountering the deaths of casualties?” and “Do you believe that these encounters have caused problems for you?” Based on the participants’ responses, follow-up questions were asked. The duration of interviews ranged from 45 to 100 min, with 10-minute breaks provided in cases where interviews exceeded 60 min. Interviews were conducted in a convenient setting. Data saturation was reached, and interviews were concluded when no new codes emerged. Tracking questions, such as “How?” and “Why?” and “Can you elaborate more on…” were asked during interviews. Furthermore, many other aspects of the interviewees’ opinions that arose during the conversation were also investigated. The interview questionnaire file is attached as an appendix.

### Data analysis

Data analysis was conducted simultaneously with data collection. All interviews were transcribed verbatim immediately after recording. Researchers took notes during the interviews. After each interview, the text was first transcribed on paper and reviewed several times to gain an overall understanding. For each interview text, a summary was written, and hidden meanings were identified. Data analysis was carried out using Smith et al., (2004, 2011) approach, which includes the following step-by-step stages: (1) Reading and rereading the text; (2) Initial notetaking; (3) Developing apparent themes; (4) Searching for connections between themes; (5) Moving to new themes; and (6) Searching for final themes and subthemes [11, 12]. Manual data analysis was employed to ensure data quality. Reviewing texts and sources occurred after data analysis was completed.

### Rigor

To ensure the reliability of qualitative data, we employed Guba and Lincoln’s criteria, including credibility, transferability, dependability, and confirmability [13].

### Ethical considerations

The project received ethical approval from the Shiraz University of Medical Sciences Research Vice-Chancellor (IR.SUMS.MED.REC.1402.081). Researchers introduced themselves to the participants and explained the objective of the research, assuring them that all information gathered during the interviews would remain confidential. Participants were selected based on their willingness to participate in the study. Additionally, participants were assured that if they wished to withdraw from any stage of the research, they could do so. Other ethical considerations included obtaining written informed consent from the participants, assuring them that research results would be made available to them upon request, maintaining the confidentiality of research unit information, and expressing gratitude to all individuals who collaborated with us in the research. Participants had the autonomy to continue interviews or participate in the study.

## Results

All 17 participants in the study were male. Participants’ mean age was 39 with a range of 24–60 and with a mean work experience of 15 years ranging from 5 to 30 years. The demographic and occupational characteristics of the participants are presented in Table 1.

**Table 1:** Demographic and occupational characteristics of the participants

Variable		Number
**Personnel**	EMS	**10**
	Red Crescent	**7**
**Education**	Associate’s Degree	11
	Bachelor’s Degree 5	5
	Master’s Degree	1
**Marital Status**	Married	13
	Single	4
**Work Experience**	5–10 years	4
	11–20 years	7
	21–30 years	6

The 17 personnel of EMS and the Red Crescent who expressed a willingness to participate in the study and had experience when encountering deaths in response to emergencies and disasters were selected through purposive sampling from urban and road bases in Fars, Isfahan, and Alborz provinces in Iran and then were interviewed. By analyzing the interviews, a total of 451 codes were extracted. By removing the duplicate codes and merging the similar ones, finally, we found the lived experiences of emergency personnel when encountering deaths in response to emergencies and disasters in Iran in the three main themes of psychological and emotional problems, mental and physical problems, personality-disposition, and behavioral problems. Sub-themes and sub-sub-themes are also provided in Table 2.

**Table 2:** Themes, sub-themes, and sub-sub-themes from interviews with participants to explain the lived experiences of emergency personnel when encountering deaths in response to emergencies and disasters in Iran

Main theme	Sub-theme	Sub-sub-theme
Psychological and Emotional status	Psychological Problems	• Depression • Stress • Boredom • Anxiety
	Emotional Problems	• Feelings of emptiness and hopelessness • Feelings of guilt • Feelings of loneliness • Becoming sensitive toward family • Becoming emotional
Personality, Disposition, and Behavior status	Personality Problems	• Neglect of personal hygiene • Withdrawal • Aggressiveness and involvement • Negative impact on interpersonal relationships
	Disposition Problems	• Immorality • Irritability
	Behavioral	• Becoming more patient • Becoming accustomed • Becoming cautious in driving
Mental and physical status	Physical problems Nutritional disorders	• Musculoskeletal pain • Digestive problems • Heart palpitations • Headache • Dizziness • Fatigue • Tremors in hands and feet • Weight gain and weight loss • Decreased appetite • Anorexia (literally, "lack of appetite") • Hyperphagia (excessive appetite)
	Sleep disorders	• Excessive sleep • Insomnia (literally, "lack of sleep") • Having nightmares
	Concentration and Memory disorders	• Decreased concentration • Mental turmoil or agitation • Flashbacks of distressing scenes • Forgetfulness

The first theme extracted from the data analysis was related to psychological and emotional status, encompassing sub-themes such as psychological and emotional problems. Depression, stress, anxiety, and boredom are included in the sub-sub theme of psychological problems. Feelings of emptiness and hopelessness, a sense of loneliness, feelings of guilt, becoming sensitive toward family, and becoming emotional are included in the sub-sub theme of emotional problems. Most of the emergency responders described their working conditions as stressful and hazardous. They believed they could endure a significant amount of stress when dealing with the deaths of the injured or those facing critical physical conditions or imminent death. They also believed that these conditions became even more challenging when dealing with injured children and pregnant mothers showing that the condition of patients is of the determining role in the stress that is conveyed to emergency service providers. In their daily lives, they experienced stress and anxiety to the extent that they reported having unexplained worries and anxieties. At times, they would lose interest in any activities, and after witnessing numerous deaths and hearing the cries of the injured and their companions, they would feel a sense of emptiness and hopelessness in life. They even thought that perhaps they could have acted better and prevented the death of an injured person, leading to feelings of guilt. What can be referred from the result of this study is that at least in the case of the participants of this study what the participants faced in the workplace affected their level of stress and psychological aspect of their life.

Participant #1 expressed his feelings of hopelessness and emptiness in the following manner: *“When I see the cries of the injured and their companions at various accident scenes*,* especially when I can’t do anything to help them*,* I feel a sense of emptiness and hopelessness. It makes me realize how fragile life can be and how close death is to all of us.”*

Participant #8 described his stress and anxiety in the following way: “*Sometimes when I’m at home and don’t have any specific tasks to do*,* I suddenly feel anxious and stressed for no apparent reason. I don’t even know why it happens*,* but it feels like something is stirring inside me. Usually*,* during such times*,* I try to keep myself busy.”*

Participant #7 expressed his anxiety as follows: *“When I’m at home*,* and the home phone or the doorbell rings unexpectedly*,* I unconsciously become anxious and stressed. Or when I’m at the station*,* and nothing significant is happening*,* I get stressed*,* so I get up and use my car to keep myself occupied.”* These descriptions highlight how these participants experience anxiety and stress in different situations and how they cope with it.

Participant #14 expressed feelings of depression and boredom, stating: *“I feel so bored and depressed; sometimes I think it might be better to see a psychologist.”*

Participant #16 described signs of depression during his interview, stating: *“I’m bored*,* I have no interest in anything*,* I feel like I lack vitality and liveliness in my personal life*,* and I constantly feel tired.”* These statements reflect the participants’ experiences of depression and its effects on their motivation, interest, and overall emotional well-being.

Participant #10 mentioned, *“Sometimes I feel so bored and lethargic*,* so that I don’t even have the motivation to take care of personal hygiene.”* This statement highlights the extent of their boredom and lack of energy, which can affect even basic self-care routines like personal hygiene.

The second theme encompassed personality, disposition, and behavior status. included sub-themes of personality, disposition, and behavior problems. Neglect of personal hygiene, withdrawal, aggressiveness, and involvement, and negative impact on interpersonal relationships are included in the sub-sub theme of personality problems, immorality, and irritability are included in the sub-sub theme of disposition problems, and becoming more patient, becoming accustomed and becoming cautious in driving are included in the sub-sub theme of behavioral. Mood and behavior, with participants describing various emotional and behavioral responses. These responses included irritability, aggressiveness, impatience, heightened emotional sensitivity, normalization of distressing situations, indifference, neglect of personal hygiene, withdrawal, caution while driving, negative impact on interpersonal relationships, and increased sensitivity towards family members. Participant no 6 mentioned that he had become desensitized to distressing incidents over time while also expressing increased sensitivity towards their family members. On the other hand, participants no 7 and no 12 stated that they had been scolded by their family members for becoming irritable and aggressive, and they reported strained relationships with authorities due to their strong reactions. This part of our data supports the fact that changes in the psychological aspects of participants’ lives can probably result in a change in the behaviors adopted by participants towards their family members.

The third theme addressed changes in mental and physical status. This theme is divided into four sub-themes. musculoskeletal pain, gastrointestinal issues, heart palpitations, headaches, dizziness, fatigue, tremors in the hands and feet, and weight fluctuations are included in the sub-sub theme of physical problems. decreased concentration, mental turmoil, flashbacks of distressing scenes, and forgetfulness are included in the sub-sub theme of concentration and memory disorders. insomnia, nightmares, and excessive sleep are included in the sub-sub theme of sleep disorders. and decreased appetite, anorexia, and hyperphagia are included in the sub-sub theme of nutritional disorders. Participants stated that, in general, they had less appetite for food compared to before their exposure to death-related incidents. Immediately after experiencing events where someone loses his/her life, they often have no appetite for food at all. In some cases, they reported instances of overeating or nervous hyperphagia, but these occurrences were limited in number. This sub-subtheme highlights how the participants’ eating habits and appetites were affected by their experiences, leading to changes in their nutritional patterns. The results showed the fact that in the case of the participants of this study, different neurophysiological systems in their bodies can be affected by external inputs that they received by facing a particularly unpleasant situation.

These sub-subthemes provide insights into various ways in which emergency responders are affected by their experiences, including emotional, behavioral, and cognitive responses, as well as changes in their eating habits and appetites.

Many participants mentioned that they struggled to focus on their daily tasks and often forgot even small and routine matters. Some also experienced flashbacks of distressing scenes from past incidents.

Participant no 17 mentioned occasional instances where previous distressing scenes came to mind when responding to a new incident. Participant no 16 described how distressing scenes would sometimes intrude into their thoughts when they were idle at home.

Participant no 7, 12, and 9 all mentioned forgetfulness as one of their problems after encountering death-related incidents. Participant no 9 specifically stated that he had excellent memory during his school years but felt that his memory had deteriorated since starting his emergency response career. He cited instances of forgetting even the smallest details that his family members asked him to purchase for the household.

Participant no 7 attributed his forgetfulness to mental turmoil and excessive mental preoccupation, which led to forgetting many trivial matters throughout the day.

Many participants complained of insomnia and delayed sleep, while some mentioned that they slept too much and often wanted to take a nap. Participants no 8 and 2 mentioned grinding their teeth during sleep, necessitating the use of dental guards for protection. This part of the data showed that even unconscious aspects like sleep and movements during sleep of a routine life which is not expected to be under the control of individuals can be highly affected by what the participants face in their workplace.

Participant no 7 stated, *“I often find myself sleeping whenever I have spare time*,* and no matter how much I sleep; I don’t feel refreshed.”* Participant no 8 mentioned having nightmares at night, sometimes waking up with a start due to the sound of explosions and screams.

Various participants described different physical issues they encountered in their daily lives. One common problem among most participants was heart palpitations, with some experiencing them constantly and others suddenly. Participant no 10 mentioned experiencing unexplained headaches and dizziness at times. This is suggested by our data that not only the psychological aspects of participants were affected by the inputs that they received in their workplace but also the physiological condition of their bodies like the level of energy was targeted.

Participants no 14 and 15 mentioned feeling excessively fatigued, with Participant no15 expressing it as *“I feel very tired; my body feels exhausted all the time.”*

This research highlights that many emergency responders who participated in this study reported a multitude of issues, including sleep disturbances, depression, stress and anxiety, communication problems with others, increased sensitivity toward family members, and the transfer of these issues to their families. It is evident that these problems have a significant impact on the personal lives and well-being of these personnel.

## Discussion

Emergency personnel who respond to incidents and emergencies are exposed to significant psychological and emotional stress due to the nature of their profession, as well as the pressure to provide optimal care to the injured. This often leads to various mental and emotional challenges, impacting both their personal and professional lives. As the results of this research have shown, nearly all participants experienced mental health issues such as stress, anxiety, depression, and burnout. These findings are consistent with those of the studies conducted on emergency personnel in various parts of the world. For instance, a study in Saudi Arabia revealed that the highest reported mental disorders among emergency responders were anxiety (%19.3) and depression (%9.2) [14]. Moreover, another study demonstrated that EMS personnel faced high levels of stress when dealing with critically injured patients [15]. In Pakistan, %22 of the study participants reported experiencing PTSD, and %28 had a history of anxiety and depression [16]. A study conducted in Spain during the COVID-19 pandemic found that female EMT personnel experienced higher levels of stress, exacerbated by the lack of personal protective equipment (PPE) and fear of contracting the virus [17]. Another study in Spain indicated that EMS personnel in the country suffered from high levels of stress, anxiety, and depression [18]. In the United States, a study conducted on 3.9 million American nurses revealed that 31.5% of them left their profession due to job-related fatigue [19]. Furthermore, other studies suggest that EMTs have a higher risk of suicide in comparison to the general population [20]. Participants in this study stated experiencing temporary and, in some cases, long-lasting nutritional disorders, such as reduced appetite, loss of appetite, and overeating, especially in incidents involving fatalities among the injured people. It was commonly reported by participants that they had little to no appetite in the initial hours after they faced the death of injured people, and in some cases, this lack of appetite was reported to persist for days. Some participants reported that whenever they tried to eat, the disturbing scenes of the deceased flashed in their minds, reducing their appetite. The obtained data from other related studies in this area are in a good harmony with our data. A study carried out on military nurses in Turkey showed that the psychological effects and depression resulting from job-related conditions caused weight loss and decreased appetite [21]. There are other researches showing that paramedics, during their service, experience a higher increase in BMI compared to others [22]. Consistent with this result, a study carried out on 134 Australian paramedics showed that 23.1% of the participants were obese, and 34.2% of them were overweight [23]. Further, another study showed that nurses who worked on the frontline during the COVID-19 pandemic experienced weight loss compared to their counterparts [24].

Participants in this study had diverse backgrounds and employment relationships. Those with more experience and greater exposure to critically injured and deceased individuals tended to perceive such experiences as more normal and did not deny the impact of these experiences on their mood and behavior. Being more nervous and sensitive in comparison to before their employment was a common report among the participants. Furthermore, participants in the current study pointed out that their families witnessed their increased sensitivity and aggression, and some participants even reported having verbal confrontations with their supervisors. Same results in this regards is reported by a study conducted during the COVID-19 pandemic showed that the stress, threats, and risks related to COVID-19 transmission to healthcare workers ultimately heightened their nervousness and aggressiveness [25].

Working as a paramedic generally involves physiological reactions and is associated with threats and challenging responses, which often require a high level of focus and concurrent execution. Cognitive difficulties, mental disorientation, and lack of focus caused by job situations were common subjects among the participants in this study. However, neither incidents of lack of concentration nor forgetfulness during missions were reported in this study, nor participants believes that they even had better concentration during operations and were wide-awake during remembering medical procedures.

Contrary to our results, in some studies experiencing of weakened concentration has been reported by participants [26, 27]. Furthermore, shift work and overnight shifts, which are common among emergency personnel working 12 or 24-hour shifts, can have significant detrimental outcomes on cognition related to concentration, memory, and responding skills [28].

Considering the sleep related problems our results are supported by other studies in this area showing that emergency personnel who generally work in 24-hour shifts, responding to emergencies and incidents around the clock; can experience various sleeping pattern interruptions because of their work condition [28]. Participants in this study reported a variety of sleep problems, such as insomnia, hypersomnia, nightmares, and bruxism. Generally, emergency personnel do not experience healthy levels of sleep quality and refreshing sleep [29]. A study involving all members of the Australian emergency department reported that 20% of participants experienced insomnia [23].

For emergency responders who work for 24 h and seven days are continuously on call and participate in high-stress missions with demanding workloads and pressure, it is necessary to retain physical and mental fitness [30]. Numerous studies have shown that some healthcare providers are at a high risk of musculoskeletal injuries, especially lower back pain, and emergency personnel are no exception [31]. Parallel with the results of previous studies, in this study also a considerable number of participants reported different physical problems, such as back pain, musculoskeletal problems, heart racing, and shaking hands and feet. In addition, in a study in New Zealand, emergency and paramedic personnel reported lower back pain and musculoskeletal problems [32]. It is worth mentioning that musculoskeletal problems are just one of the physical issues that can be potentially faced by emergency personnel. In another relevant study, more than 88% of emergency personnel also complained about one of the risk factors for cardiovascular problems [33].

Given the heavy workload and stressful nature of emergency responders’ jobs, they are exposed to numerous risks threatening their mental, physical, and emotional well-being. The issues that emergency personnel participating in this study reported included forgetfulness, insomnia, stress, fatigue, hopelessness, back pain, and heart racing.

### Limitations

In this study, some of the emergency personnel from EMS and Red Crescent bases who experienced encounters with the deaths of casualties during their service participated. The process of identifying individuals who met these criteria and were willing to be interviewed was time-consuming. Additionally, there were individuals who met the criteria for participation but were not willing to do interviews. Another limitation of this study was the inability to access female emergency personnel, as all the participants in this study were male emergency personnel which can make a gender specific result.

### Suggestions

As previously mentioned, this study focused on male emergency personnel present at emergency bases, and all the participants were male. Therefore, conducting a similar study on female emergency personnel and comparing the results obtained from these two studies would be valuable and could contribute to providing better care services for emergency personnel. Additionally, conducting a study using different measurement methods to assess the impact of various types of deaths, including the death of a colleague, death of male and female casualties, as well as children and the elderly, on the mental and physical health of these personnel is recommended.

## Conclusion

This research presents a sensible viewpoint on a relatively underexplored topic, the lived experience of emergency personnel with a history of encountering the deaths of casualties during their missions. This study has shown that emergency personnel are highly susceptible to various physical, psychological, and emotional injuries because of encountering the deaths of casualties. These injuries have the potential to exert an impact on their lives, making them more vulnerable compared to the general population. It is recommended that policymakers and clinical educators make ways to prevent these problems and provide emergency personnel with physical, psychological, and emotional support.

### Electronic supplementary material

Below is the link to the electronic supplementary material.

Supplementary Material 1

## Data Availability

The datasets used and/or analyzed during the current study are available from the corresponding author on reasonable request.
