# Supplementary material for: Exploring paramedics’ lived experiences in confrontation with patients’ death during missions: a phenomenological study
Source: BMC Emerg Med. 2024 Jul 12;24:115. doi: 10.1186/s12873-024-01042-6 (PMC11241868; doi:10.1186/s12873-024-01042-6)
Supplement: Supplementary file 1 — Supplementary Material 1 [file 12873_2024_1042_MOESM1_ESM.docx]

| **Semi structured Interview Questionnaire**  **Last Name: First Name: Education:**    **Working Experience (Years):**  **Age: Gender: Code:** |
| --- |
| 1. How does facing a patient's death impact your emotional well-being? 2. Have you noticed any changes in your behavior and attitude? 3. Does encountering a patient's death affect your appetite? 4. Have you experienced any changes in your physical or psychological well-being? 5. Are there any consequences related to facing a patient's death that influence your relationships, both with others and your family? 6. Do you believe that dealing with the death of patients can be a factor in changing your motivation? 7. Do you think facing the death of patients might alter your attitudes towards raising children, if applicable? 8. Have you ever reflected on the memories of a patient's death in your mind or shared them with others? 9. Have you ever exhibited unacceptable behavior or reactions that led to admonishment from your family? 10. Has your memory or concentration been impacted by the death of a patient? 11. Please include any additional points you consider relevant to the topic. |
